# Seroprevalence and risk factors of West Nile virus infection in veterinarians and horses in Northern Palestine

**DOI:** 10.14202/vetworld.2021.1241-1246

**Published:** 2021-05-21

**Authors:** Ibrahim Alzuheir, Adnan Fayyad, Nasr Jalboush, Rosemary Abdallah, Sameeh Abutarbush, Mohammad Gharaibeh, Majd Bdarneh, Nimer Khraim, Mohammad Abu Helal, Belal Abu Helal

**Affiliations:** 1 Department of Veterinary Medicine, An-Najah National University, P.O. Box 7 Nablus, Palestine; 2 Palestinian Livestock Development Center, Tubas-Palestine; 3 Department of Clinical Veterinary Medical Sciences, Faculty of Veterinary Medicine, Jordan University of Science and Technology, P.O. Box 3030 Irbid, 22110 Jordan; 4 Department of Basic Veterinary Medical Science, Faculty of Veterinary Medicine, Jordan University of Science and Technology, P. O. Box 3030 Irbid, 22110, Jordan; 5 Department of Public Health Sciences, Faculty of Graduate Studies, An-Najah National University, P.O. Box 7 Nablus, Palestine

**Keywords:** enzyme-linked immunosorbent assay, *Flavivirus*, horses, Palestine, veterinarians, West Nile virus

## Abstract

**Background and Aim:**

West Nile fever (WNF) is a neurotropic, mosquito-borne disease affecting humans and domesticated animals, caused by a member of the genus *Flavivirus*. Over the last decades, this virus has been responsible for several cases of illness in humans and animals. The current epidemiological status of WNF in horses is insufficient, and in veterinarians, as an occupational hazard is unknown. This study aimed to investigate and determine the seroprevalence and risk factors for WNF in veterinarians and horses in Palestine.

**Materials and Methods:**

In this study, serum samples from 100 veterinarians and 87 horses were collected between August 2020 and September 2020 from different cities of Northern Palestine. West Nile virus (WNV) antibodies were detected using an enzyme-linked immunosorbent assay.

**Results:**

Our results showed that 60.9% of the horse serum samples were positive in all investigated cities. In horses, location is a risk factor for the seropositivity for WNF, whereas age, sex, breed, and intended use of the horses, were not associated with increased WNF seropositivity. In veterinarians, 23.0% of the serum samples were positive. Positive samples were detected in all locations, age groups, experience length, and work sectors. However, the seropositivity for WNF was not influenced by these variables.

**Conclusion:**

The results revealed that WNV circulates in most regions of Palestine. Our results will help determine the risk of infection in animals and humans and control WNV transmission. Surveillance studies on humans, vectors, and animals are needed to better define endemic areas.

## Introduction

The genus *Flavivirus* causes important zoonotic, neurotropic, and arthropod-borne diseases in humans and domesticated animals. This genus includes West Nile virus (WNV), Japanese encephalitis virus (JEV), and tick-borne encephalitis virus (TBEV) [1]. The principal vector of WNF and JEV is mosquitoes of the genus *Culex*, whereas TBEV transmission is by ticks of the genus *Ixodes* [2]. The diseases induced by these organisms are in the World Organization for Animal Health (OIE) list as notifiable diseases. Humans, birds, horses, and other animals are the hosts for WNV. The disease transmission occurs through mosquitoes that feed on virus-infected birds or wild animals.

In humans, most infections (approximately 80%) were asymptomatic [3]. After an incubation period of 2-14 days, approximately 20% of infected individuals develop self-limiting flu-like symptoms for 3-6 days. Only approximately 1% of infected persons fall seriously ill with neurological symptoms (mainly meningitis and encephalitis) [3]. Veterinarians have a higher risk of developing zoonotic diseases than other groups of people and professions. A veterinarian can become infected through indirect contact with an infected animal, especially birds, or exposure to mosquitoes when located in a high-risk area [4]. In horses, West Nile fever (WNF) clinical signs include fever, ataxia, behavioral changes, and paresis or paralysis. Approximately 10% of affected horses show neurological disorders compared with 1% of infected humans. In horses, the clinical signs of WNF are difficult to differentiate from those of other diseases, leading to the misdiagnosis of WNF [5]. Various determinants are associated with the transmission and circulation of WNV, including the interactions between the virus, vector, host, and environment. Wild and migratory birds can maintain high viral titer in their blood, which, therefore, plays an important role in the transmission of viruses. However, humans and horses are considered dead-end for the virus circulation as viremia is not maintained for mosquito transmission [6]. Climate change (mainly temperature, humidity, and rainfall) impacts migratory birds and vector distribution. Therefore, continuous monitoring of WNF in vectors, birds, animals, and humans is required [7]. The location of Palestine (between three continents, i.e., Asia, Africa, and Europe) has made it the locus of a crossroad for bird migration [8].

The last and the only study on WNF in domesticated animals in Palestine was conducted in 2014 [9]. The occurrence and risk factors of WNF in veterinarians as an occupational hazard have not been investigated. Thus, this study aimed to investigate WVF and to determine the seroprevalence and risk factors for WNV in veterinarians and horses in Palestine.

## Materials and Methods

### Ethical approval

All experimental procedures performed were approved by the Institutional Review Board of An-Najah National University and the scientific research committee of the Master of Public Health Program as well as the faculty of graduate studies scientific research board council at An-Najah National University with archive number: (5) Nov. 2019.

### Study period and location

All samples were collected in August and September 2020 and analyzed in November 2020. Cities in Northern Palestine were targeted in this study. The number of registered veterinarians in the target areas is 159 (Veterinarian Association, Jerusalem Branch). One hundred blood samples were obtained from veterinarians working in four cities of Palestine (Jeninn=29) (32°27′40″N 35°18′00″E), (Nablus n=22) (32°13′20″N 35°15′40″E), (Tulkarm n=31) (32°18′42″N 35°01′38″E), and Qalqiliya (n=18) (32°11′25″N 34°58′07″E).

**Figure-1:**
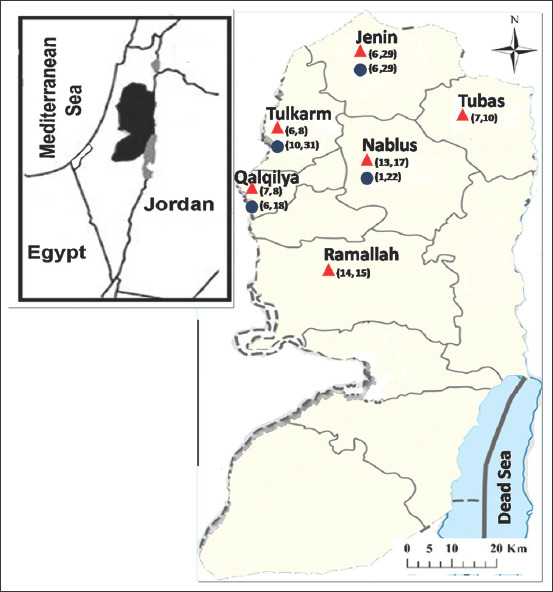
Map of Palestine. Areas where the samples were collected. Numbers indicated by a red triangle and blue circle for horses and veterinarians positive sample from collected samples, respectively [Source: https://www.gif-map.com/maps-of-asia/maps-of-west-bank].

The horse population in Northern Palestine, as reported by the latest statistical data from the Palestinian Central Bureau of Statistics, is estimated to be 1835 [10]. For horses, 87 blood samples from five cities of Northern Palestine (Jenin, n=30; Nablus, n=23; Tulkarm, n=8; Tubas, n=10; and Qalqiliya, n=18) (Figure-1) were obtained.

### Sample collection and organization of data

#### Veterinarians

One hundred veterinarians participated in this study. Each veterinarian provided their consent for participation in the study and filled out a questionnaire, consisting of questions on age, experience length in years (1-5, 6-10, 11-15, 16-20, and >20 years), work nature (administrative or field), and work sector (private or public). Venous blood collection was performed under standard conditions by a trained nurse. The standard blood sample volume was 3-5 mL and was collected using a sterile syringe from a peripheral vein in the antecubital fossa after wiping with 70% ethanol. The samples were placed in a plain tube and then transported in an icebox to the Virology Laboratory at the Department of Veterinary Medicine, An Najah National University. The samples were placed inside a refrigerator for approximately 2-4 h for the clot to be fully formed. Serum was obtained by centrifugation at 3000 rpm for 10 min at room temperature. After centrifugation, the serum was secured at –20°C until being processed.

#### Horses

Eighty-seven horses were included in this study. For each horse, a questionnaire consisting of questions on location, age in years (<5, 5-10, and >10 years), sex, breed, and use of the horse was filled. Available data and samples from Ramallah (n=15), which is located in the middle of Palestine, were also included. Five-milliliters blood was collected in a plain tube through venipuncture to the jugular vein using a sterile needle syringe directly after clinical examination by a veterinarian. The blood samples were allowed to clot fully, and serum was separated and stored as described previously.

### Enzyme-linked immunosorbent assay (ELISA)

Sera were screened for WNV-specific antibodies using a commercially available competition ELISA, which allows the species-independent recognition of WNV IgG antibodies against precursor membrane and envelope proteins (ID Screen^®^ West Nile Competition, IDVet, Montpellier, France). According to the manufacturer, the monoclonal antibody used in this kit cross-reacts with Japanese encephalitis and TBEVs. The ELISA cutoff value is defined by the residual binding ratios of the sample (S) to the negative control (N) (S/N% value); sera with S/N ratios of 40% and lower are positive, whereas those with S/N ratios of more than 50% are considered WNV antibody-negative. S/N values of 40-50% are inconclusive. A virus neutralization test revealed a specificity of 89.5% and sensitivity of 88% [11].

### Statistical analysis

The associations between WNF seropositivity and selected characteristics were tested using the Chi-square test. p<0.05 was used to denote statistical significance, and all statistical analyses were performed using Statistical Package for the Social Sciences, version 21 (IBM Corp., Armonk, NY, USA).

## Results

### Veterinarians

The examinations of 100 serum samples from veterinarians from four cities of Palestine revealed WNF seropositivity of 23.0%. WNF antibody was detected among all locations, age groups, experience length, and work sectors (Table-1). All involved veterinarians were healthy at the time of blood collection and had no history of central nervous system infection, WNF, or any *Flavivirus* vaccination. The Chi-square test revealed that all these variables were not risk factors for the seropositivity for WNF (p>0.05).

**Table-1:** Univariable analysis factors associated with seropositivity in veterinarians to WNV in Northern Palestine 2020.

Variable	Category	WNV. Ab. No. of +ve (Total)	WNV Ab positive %	p-value
Location	Jenin	6 (29)	20.7%	0.075
	Nablus	1 (22)	4.5%	
	Qalqilya	6 (18)	33.3%	
	Tulkarm	10 (31)	32.2%	
Age	24-29	10 (40)	25.0%	0.842
	30-35	4 (25)	16.0%	
	36-40	2 (10)	20.0%	
	41-45	1 (5)	20.0%	
	>45	6 (20)	30.0%	
Work period	1-5	10 (37)	27.0%	0.862
	6-10	4 (20)	20.0%	
	11-15	2 (15)	13.3%	
	15-20	2 (8)	25.0%	
	>20	5 (20)	25.0%	
Work sector	Public	9 (34)	26.5%	0.362
	Private	14 (66)	21.2%	
Work type	Administrative	2 (11)	18.2%	0.514
	Field	21 (89)	23.6%	

WNV: West Nile virus

#### Horses

Horse sera from 87 animals kept in 6 West Bank cities in Palestine were collected in 2020 (Figure-1 and Table-2). All involved horses were healthy at the time of blood collection and had no history of central nervous system infection, WNF, or any *Flavivirus* vaccination. The overall prevalence rate was 60.9%, ranging from 20.5% to 93.3%. WNV antibody-positive animals were detected in all investigated cities, ages, sex, and uses. High prevalence rates were detected in Ramallah and Qalqilya (93.3% and 87.5%, respectively). The lowest prevalence rate was detected in Jenin (20.7%). The number of antibody-positive animals increased as age increased; however, this difference was not statistically significant. The Chi-square analysis revealed that location was the only risk factor for the seropositivity for WNF (p<0.001). Age, sex, use, and breed were not associated with the increased risk of seropositivity for WNF (p>0.05) (Table-2).

**Table-2:** Univariable analysis factors associated with seropositivity in horses to WNV in Northern Palestine 2020.

Variable	Category	WNV. Ab. No. of +ve (Total)	WNV Ab. % of +ve	p-value
Location	Jenin	6 (29)	20.7%	<0.001*
	Nablus	13 (17)	76.5%	
	Qalqilya	7 (8)	87.5%	
	Ramallah	14 (15)	93.3%	
	Tulkarm	6 (8)	75.0%	
	Tubas	7 (10)	70.0%	
Age	1-5	26 (47)	55.3%	0.501
	5-10	20 (30)	66.6%	
	>10	7 (10)	70.0%	
Sex	Male	30 (47)	63.8%	0.351
	Female	23 (40)	57.5%	
Use	Racing	18 (28)	64.3%	0.156
	Pleasure riding	14 (25)	56.0%	
	Jumping	2 (7)	28.6%	
	Show	19 (26)	73.1%	
	Riding	0 (1)	0.0%	
Breed	Arabian	34 (59)	57.6%	0.318
	Thoroughbred	2 (3)	66.6%	
	Belgian	0 (2)	0.0%	
	Cross	3 (5)	60.0%	
	Local	6 (7)	85.7%	
	ND	8 (11)	72.7%	

* Factors statistically significant at p≤0.1. WNV: West Nile virus

## Discussion

WNF is a mosquito-borne disease. The *Culicidae* family is the most common vector of the virus to spread the disease [12]. The availability of *Culicidae* spp. and wild birds in Palestine and studies on WNF support the conditions of the virus circulation [13]. Besides, WNF can be transferred by contamination with infected blood and blood components, tissues, and cells and organ transplantation [14]. Following studies on WNF in domesticated animals, this is the first study on the seroepidemiology of WNF in veterinarians in Palestine. No vaccine is available in Palestine, and all seropositive samples resulted from exposure to the virus. Competitive indirect ELISA for detecting IgG is a useful tool for screening previous exposure to the virus from 10 days to several years after exposure [15]. Antibody cross-reactivity of ELISA with other viruses of the genus *Flavivirus*, including JEV and TBEV, has been reported [16]. However, this cross-reactivity could be advantageous in controlling emerging *Flaviviruses* because it ensures partial cross-protection [16]. TBEV and JEV are not endemic in Palestine and Israel [9]. However, further confirmatory tests are required to exclude cross-reactivity.

WNF is considered an occupational hazard disease. A study has indicated laboratory-acquired infections with WNV through percutaneous inoculation [14]. Another study from the US has indicated a 57% (n=90) IgM seropositive rate in turkey farmers; six reported febrile illness [17]. Veterinary is a high profession risk of WNF [18]. A veterinary student could be infected after an autopsy of an infected horse, and the most likely route of infection was through mucous membrane droplet [19]. Data regarding the prevalence of WNF in veterinarians or animal healthcare providers are rare. In this study, a 23.0% (n=100) seroprevalence rate was detected in veterinarians. This rate is higher than non-occupational hazard prevalence rate in Jordan (8%; n=261), indicating that WNF is not related to sex, age, or interaction with domesticated animals [20]. A study in Morocco has reported a 5.2% (n=250) seroprevalence rate [21]. The seroprevalence rate in healthy blood donors was 0.9% (n=2821) in Turkey [22] and 1.2% (n=864) in Italy [23]. Compared with these rates; the high prevalence of veterinarians in Palestine highlights the occupational hazard of WNF. Our findings showed that WNF was not associated with location, age, work period, work sector, or work type of the veterinarians. This might be due to the endemic state of WNF in Palestine [9]. The data obtained from horse WNV could be used to raise awareness among public and veterinary health experts and to trigger an enhanced surveillance and prevention activities [24]. In horses, the overall seroprevalence was 60.9% (n=87), which is consistent with those from previous WNF seroprevalence studies in horses in Palestine in 2014 (73%, n=210) [9], indicating that WNF is cycling in Palestine. This rate lies between the prevalence rates in endemic regions, such as South Africa (75%, n=243) [25] and Chad (97%, n=30) [26], and those detected in neighboring countries, such as Jordan (24.9%, n=253) [27] and Egypt (20.7%, n=400) [28], and in European countries, such as France (8.5%, n=432) [15]. Despite the high seroprevalence rate of WNF in horses, no clinical or laboratory diagnosis has been reported in Palestine. This might be due to the lack of knowledge about the disease, unavailability of commercial tests, unfamiliarity of veterinarians to the disease, and absence of a veterinary service reporting system. The difference in seroprevalence rates in horses was correlated with the geographical area. A study on horses in Palestine has shown that Jenin City has the lowest seroprevalence rate [9]. Previous studies [9,29] explained the higher land surface temperature related to the lower relative humidity and lower relative humidity indicated a less appropriate environment for mosquito survival and blood retrieval success risk factors for the disease. Horses in other cities appeared to be at higher risk of developing WNF. In agreement with our findings, a study conducted in Palestine has indicated that the seroprevalence rate is higher in central and southern cities, where the climate is less appropriate for mosquito survival [9]. Despite the increase in seroprevalence rate with age, this difference was insignificant. Similar findings were obtained in France [15] and Jordan [27]. However, a study in Palestine (n=460) has indicated that the seroprevalence rate increased with age [9]. In this study, we tested a small number of samples and conducted a serological survey without performing a serum neutralization test (SNT). Similarly, sex and breed had no effect on seropositivity for WNV. These findings are similar to those in the studies in Jordan and France [15,27] and different from the studies in Palestine and Qatar, which have indicated that it is more likely to detect antibodies against WNV in samples collected from Thoroughbred than in samples collected from other breeds [30]. Further study involving a larger sample size and further analysis using confirmatory tests is needed to evaluate these factors.

## Conclusion

The serological detection of WNV antibodies using ELISA provides follow-up evidence that WNV could be carried by resident humans and horses in Palestine. However, ELISA cannot define the specificity of samples that may contain antigenically cross-related *Flaviviruses*, such as TBEV or JEV. In this study, antibodies against WNV were detected in the sera obtained from veterinarians and horses from different Palestinian regions, which provide evidence of the circulation of WNV in the country. This study had some limitations. First, a small number of samples were tested. Second, a serological survey was conducted without performing an SNT. The high seroprevalence rate of WNF indicates an existing infection occasion. WNF should be considered a hazard for specific occupational professions and a public health concern. Physicians and veterinarians should consider related clinical symptoms in exposed workers and animals and promptly report suspected cases. Surveillance studies on animals, humans, and vectors are needed to better define areas where WNF and other mosquito-borne diseases are endemic. The application of one health approach by reconnecting public health and occupational health and safety may truly improve the health of the general public, working population, and animals.

## Authors’ Contributions

IA and AF: Designed, planned, and drafted the manuscript. NJ and RA: Contributed to the ELISA work. NK and MB: Contributed to blood collection from the horses. BAH and MAH: Contributed to data and sample collection from the veterinarians. SA and MG: Contributed to statistical analysis and revised the manuscript. All authors revised and approved the final manuscript.
